# The longitudinal associations between change in physical activity and cognitive functioning in older adults with chronic illness (es)

**DOI:** 10.1186/s12877-021-02429-x

**Published:** 2021-09-04

**Authors:** Esmee Volders, Renate H. M. de Groot, Catherine A. W. Bolman, Lilian Lechner

**Affiliations:** 1 Faculty of Psychology, Open University of the Netherlands, 6419 AT Heerlen, the Netherlands; 2 Faculty of Educational Sciences, Open University of the Netherlands, 6419 AT Heerlen, the Netherlands; 3 Nutrition and Translational Research in Metabolism (School NUTRIM), Maastricht University, 6200 MD Maastricht, the Netherlands

**Keywords:** Cognition, Physical activity, Ageing, Chronic illness, Older adults

## Abstract

**Background:**

Regular physical activity (PA) is potentially beneficial for age-related cognitive decline. Although moderate-to-vigorous physical activity (MVPA) is mostly advised, older adults with chronic illnesses might benefit more from light physical activity (LPA), as they suffer from mobility problems, pain, and fatigue, limiting high-intensity PA. Therefore, the longitudinal association between change in LPA and MVPA and the change in cognitive functioning (CF) is investigated in older adults with chronic illnesses.

**Methods:**

In total 432 older adults (mean age 73.7 [±6.1] years; 46.8% female) with at least one chronic illness participated in this longitudinal observational study. Longitudinal associations between accelerometer-assessed change in PA (LPA and MVPA) and change in CF, measured with an objective validated neuropsychological test battery, were tested with multivariate linear regressions.

**Results:**

An increase in LPA between baseline and 6 months follow-up was significantly associated with improved short-term verbal memory and inhibition over the first 6 months. In addition, the change score in LPA over the first 6 months was predictive for the change score in short-term verbal memory over 12 months. Furthermore, an increase in MVPA between baseline and 6 months follow-up was significantly associated with a decrease in longer-term verbal memory scores over the same six-month period.

**Conclusions:**

For older adults with chronic illnesses who may experience difficulties in being sufficiently active, an increase in LPA is probably more achievable than an increase in MVPA. In addition, an increase in LPA enhances CF more than an increase in MVPA does.

**Trial registration:**

Netherlands Trial Register NL6005; Date of Registration 21-03-2017.

**Supplementary Information:**

The online version contains supplementary material available at 10.1186/s12877-021-02429-x.

## Background

Cognitive decline can impair the quality of life of older adults and reduce their independence [1]. Older adults with chronic illnesses are especially prone to have lower levels of cognitive functioning (CF) compared to healthy older adults [2]. Regular physical activity (PA) has been argued as an important protective factor against age-related cognitive decline, with PA at a moderate-to-vigorous intensity mostly being advised [3–5]. However, older adults with chronic illnesses, such as with osteoarthritis and cardiovascular diseases, may face difficulties in being sufficiently active due to mobility problems, pain, and fatigue [6]. Therefore, performing activities at a lighter intensity is more achievable for older adults with chronic illnesses. However, only few studies have investigated the relationship between light physical activity (LPA) and CF [7–9], and the study by Stubbs et al. [9] was the only one researching the longitudinal association. Hence, the longitudinal association in older adults with chronic illnesses between change in both LPA and moderate-to-vigorous physical activity (MVPA), on the one hand and the change in CF, on the other hand is investigated.

PA can be assessed subjectively with self-report questionnaires or objectively with accelerometers, and it can be categorised into different intensity levels: sedentary, low, moderate, and vigorous [10]. Examples of LPA activities are walking at a low speed and light household chores. Bicycling at a low speed, vacuuming, and walking briskly are examples of moderate-intensity PA. Running, carrying heavy loads, and swimming laps are examples of vigorous-intensity PA [11]. The effects of PA on physical health outcomes can be different at different intensity levels. Up to a decade ago, most research investigating the physical health benefits of PA relied mainly on self-reported PA and often did not make a distinction between PA intensities, nor was LPA considered [12]. However, people’s ability to recall PA of moderate-to-vigorous intensity is much more accurate than that of light intensity [10]. Currently, guidelines prescribe at least 150 min of MVPA spread over preferably multiple days per week to achieve physical health benefits [13, 14], such as lower risk for obesity, cardiovascular disease, some types of cancer, osteoporosis, and premature death, while mostly overlooking the role of LPA [15]. However, more recent evidence from studies assessing PA with accelerometers proved that LPA can have physical health benefits too [12, 16]. These studies suggest that LPA is inversely associated with all-cause mortality risk and associated favourably with some cardio-metabolic risk factors, including waist circumference, triglyceride levels, insulin, and presence of metabolic syndrome.

Next to the physical health benefits of PA, the evidence for cognitive health benefits of PA grows. PA can promote cognitive brain health, defined by the US Centres for Disease Control and Prevention [17] as an ability to perform all the mental processes of cognition, including the ability to learn and judge, use language, and remember, and it can counteract many effects of cognitive ageing [3, 5, 18]. The association between PA and CF has been confirmed in both cross-sectional and longitudinal cohort studies [19, 20]. However, evidence from studies regarding the effect of PA interventions on CF in older adults is inconsistent [21]. Some meta-analyses have found moderate cognitive improvements as a result of PA interventions in older adults [4, 18, 22, 23]. Yet, other meta-analyses demonstrated little to no cognitive improvements [24, 25], even when the intervention led to objectively measured increased fitness and PA behaviour [26]. Almost all meta-analyses included studies that did not take into account the actual effect of the PA intervention on PA behaviour in daily life or cardiorespiratory fitness. Thus, besides the wide variety in interventions (different types of PA activities, duration and frequency of the sessions, and the duration of the programme), one of the possible explanations for this discrepancy can be found in the many different ways in which PA was taken into account in these studies [27].

In line with research into the physical health benefits of PA, research into the cognitive health benefits of PA have also mainly focused on MVPA. When looking at effects of MVPA on CF, the executive functions (inhibition, shifting, and updating) seem to benefit the most [18, 23]. Executive functions are higher-order cognitive processes that are necessary to control cognitive behaviour. Nonetheless, studies so far have focused less on the relation of PA on a lower intensity level with CF, but it appears that LPA could also be beneficial for CF [28–30]. In recent studies, LPA has been positively associated with shifting, word fluency, processing speed, and a reduced rate of cognitive ability decline [7–9]. However, until now there is little information on whether LPA influences different aspects of CF than MVPA does.

Despite the promising benefits of PA as described above, older adults are the least physically active age group, especially when they suffer from chronic illnesses [31, 32]. Fatigue and pain are examples of PA-related barriers experienced by older adults with chronic illnesses that may result in these low levels of PA [6, 31]. Increasing PA in general, especially through MVPA, is often difficult, and it is sometimes accompanied by risks of injury and deterioration because of physical complications. Furthermore, increasing LPA is probably easier and safer for older adults. Therefore, it would be justified to determine which intensities of PA are associated with CF. Taking into account the fact that LPA (i.e., light housework, slow walking) is the dominant type of PA in older adults, especially in those who suffer from chronic illnesses, and that few of these older adults participate in meaningful amounts of MVPA, it is crucial to determine how changes in both LPA and MVPA are related to change in CF.

Randomised controlled trials are one of the best methods to test intervention effects in general and, more specifically, the effects of PA on CF. In a previous study, we tested the cognitive effects of a computer-tailored PA stimulating intervention, which consisted of three times personalised PA advice within 4 months delivered by mail and online, for older adults with chronic illnesses. We hypothesised that increasing PA would lead to improved CF. However, no intervention effects on CF were found six and 12 months after baseline [33]. The most likely explanation for this null finding was that the intervention did not lead to more objectively measured PA in this population [34].

Even though our intervention had limited effects on PA behaviour and no effects on CF, it is relevant and scientifically valuable to investigate whether and how the change in PA, operationalised as MVPA as well as LPA, is related to a change in CF in older adults with chronic illnesses, independent of the intervention. A powerful aspect of the current study is the objective measurement of PA by accelerometers on different time points and thereby taking LPA into account. We hypothesise that participants who increased their PA (i.e., between baseline and 6 months follow-up, between 6 months follow-up and 12 months follow-up, and between baseline and 12 months follow-up) showed more progress on the CF tests than those who did not increase their PA. Furthermore, we hypothesise that associations between change in PA and change in CF are expected to be stronger when considering similar time periods, as stated above, in comparison to the consideration of different, non-parallel time periods (change in PA between baseline and 6 months follow-up in relation to change in CF between baseline and 12 months follow-up). The main argument behind this hypothesis is that potential associations can fade away over time. However, it can take some time to establish a lasting change in CF. It is presumed that these changes are due to improved vascularisation, facilitation of synaptogenesis, decreased systemic inflammation, and reduced disordered protein deposition, and these lasting changes do not take place over-night [35]. Because different aspects of CF can respond differently to PA [18, 29], we analyse the associations between change in PA (LPA and MVPA) and change in different aspects of CF. The selected aspects of CF are verbal memory, shifting, inhibition, and information processing because these functions are known to deteriorate with age and can possibly improve with increased PA [19, 26, 36–39].

## Methods

### Study design and population

The present study on the association of changes in LPA and MVPA over six and 12 months, respectively, with changes in CF outcomes over the same period was part of the Active Plus and Cognitive Functioning project [40]. This project concerned a clustered two-group randomised controlled trial with a waiting list control group with assessments at baseline, 6 months, and 12 months, focused on the effect of the Active Plus intervention on CF. Intervention group participants received three times computer-tailored PA stimulating advice within 4 months (i.e., at baseline, after 2 months, and after 3 to 4 months). The online- and print-delivered advice were tailored to the specific needs and wishes of the participant and focused on incorporating PA in daily life. Data of all participants (both intervention group and control group) who completed the randomised controlled trial were used in the present study. Ethical approval for the study was obtained from the Research Ethics Committee (cETO) of the Open University, and the trial is registered in the Dutch Trial Register (protocol no. NL6005). An elaborate explanation of the study protocol was published elsewhere [40].

Six hundred and twenty-three participants were recruited from seven municipalities, which randomly invited between 500 and 4000 independently living adults aged 65 years or older living in a specific neighbourhood through an invitation letter via post. The participants met the following criteria (checked by a self-report questionnaire and a phone call with the researcher): 1) aged 65 years or older; 2) fluent in the Dutch language; 3) suffering from at least one self-reported chronic illness that affects mobility (e.g., chronic obstructive pulmonary disease, osteoarthritis, chronic heart disease) or other physical problems (e.g., visually or hearing impaired) that may affect mobility; 4) no self-reported severe cognitive problems; and 5) no wheelchair use. All participants provided written informed consent.

### Procedure

At baseline and at six and 12 months, the following procedure was adhered to: PA was assessed with an accelerometer (ActiGraph GT3X-BT) placed on the participants’ right hip for seven consecutive days prior to the CF tests. The CF tests were conducted by a trained researcher or student at the participants’ home. Inquisit 5 software [41] was used on a tablet (iPad Air 2) to execute the CF tests. The CF tests started with the first part of the Verbal Learning Test (VLT), followed by the Trail Making Test (TMT) parts A and B, the Stop-Signal Task (SST), the Letter Digit Substitution Test (LDST), and the second part of the VLT. After completing the CF tests, participants received a questionnaire to fill out within 2 weeks. The questionnaires were used to gather information on demographic variables, but also on concepts that are outside the scope of this study (e.g., self-reported PA, self-reliance, health-related quality of life). The four-month intervention started directly after completing the baseline measurement.

#### Outcome measures

##### Cognitive functioning

Table 1 provides an overview of all outcome measures. The aspects of CF (e.g., verbal memory, task switching, inhibition, processing speed) assessed in this study are chosen because they are known to deteriorate with age and can possibly improve with increased PA (Table 1) [19, 26, 36–40].

**Table 1:** Outcome measures^b^

Measurement Instrument	Concept	Measure	Scoring/ missing items	Scoring range	Higher score indicates	% valid ^**a**^
***Primary outcome measures***						
VLT	Verbal memory	Learning curve ratio	(Trial 1 + (Trial 2-Trial 1) + (Trial 3-Trial 2) + (Trial 4-Trial 3) + (Trial 5-Trial 4)) / 5	0–3 words per trial	Better learning capacity	98%
		Mean number of recalled words trial 1–5	(Trial 1 + Trial 2 + Trial 3 + Trial 4 + Trial 5) / 5	0–15 words	Better short-term verbal memory	
		Number of words recalled in delayed trial		0–15 words	Better long-term verbal memory	
TMT	Task switching	Time to complete part B minus time to complete A in sec		0-∞ sec	Worse shifting capacity	96%
SST	Inhibition	SSRT in ms	The SSRT is estimated in accordance with De Jong et al. [42] and Tannock et al. [43]. Negative SSRT values are excluded from the analyses [44].	0–1500 ms	Worse inhibition	90%
LDST	Processing speed	Number of correct substitutions		0–125 subs	Better processing speed	96%
***Physical activity***						
ActiGraph GT3X-BT	PA	MVPA minutes per week	Data downloaded with frequency extension on with ActiLife software [45]. Valid if worn 4 days during 10 h or more [46]. Non-wear definition by algorithm of Choi et al. [47]. PA scoring by Freedson-VM cut-off points [48] and by Aguilar-Fariaz cut-off points [49].	0–6720 min	More MVPA	96%
		LPA minutes per week		0–10,080 min	More LPA	

*Abbreviations*: *VLT* Verbal learning test, *TMT* Trail making test, *SST* Stop-signal task, *LDST* Letter digit substitution test, *PA* Physical activity, *SSRT* Stop-signal reaction time, *MVPA* Moderate-to-vigorous physical activit, *LPA* Light physical activity. ^a^Test outcomes were excluded if scores were deemed invalid by test administer when 1) technical problems occurred, 2) participants refused to complete a test or lacked motivation, 3) participants had physical limitations (arm amputated, hearing loss, etc.) or cognitive restrictions (participant is unable to understand the instruction), or 4) participants deviated from the instructions. ^b^Derived from Table 1 in [33]

In the VLT [37, 50], which assesses verbal memory, 15 common monosyllabic words representing concrete objects were presented one by one on an iPad screen in fixed order, with a presentation time of 1 s and an interstimulus interval of 1 s. Afterwards, participants were asked to verbally recall the words they had remembered. The first trial was followed by four more trials in which the words were presented in identical order and each followed by an immediate free recall procedure. After a delay of 15–25 min in which the remaining CF tests were assessed, and unexpectedly for the participants, the instruction was given to recall the 15 words learned once more. Finally, a recognition trial was administered where participants had to recognise the 15 learned words out of 30 words. Outcome measures for the VLT were the learning curve ratio over trials 1–5, the mean number of recalled words in trial 1–5, and the number of words recalled in the delayed trial (Table 1).

During the TMT parts A and B [51], which can be used to assess task switching when both parts are administered, participants had to draw lines with their fingers on an iPad screen connecting 25 randomly placed numbers in the correct order (part A) or numbers and letters alternatively (part B). Both parts A and B were preceded by a practice trial. The time in seconds required to complete the task was noted for each task. The outcome measure task switching was operationalised as the time to complete part B minus the time to complete part A [52].

In the SST [53], which is an inhibition task, participants had to quickly press the left-hand button if the arrow on the iPad screen pointed to the left and press the right-hand button if the arrow pointed to the right. However, when a signal beep was played after the presentation of the arrow, participants had to inhibit their reaction and withheld from pressing either of the buttons. These beeps occurred in 25% of the trials. Firstly, participants could practice the task in a block of 32 trials. Afterward, three blocks of 64 trials were completed with 10 s of rest in between blocks. The stop-signal delay between presentation of the arrow and signal beep was varied and depended on participants’ performance. The delay, which started at 250 milliseconds (ms), was increased by 50 ms if the previous inhibition was successful. The delay got shorter by 50 ms if the previous inhibition was unsuccessful. This stop-signal delay staircase design ensured that participants were able to inhibit their response on approximately half of all trials. The inhibition outcome measure was operationalised as the stop-signal reaction time in ms (SSRT).

During the LDST [38], which is a processing speed task, participants were presented with a matrix. Odd rows contained letters; even rows contained empty answer boxes. The task was to translate the letters by clicking the corresponding digits with the help of a provided key. After a practice round of 10 letters, the participant had 60 s to replace as many randomised letters with the appropriate digit indicated by the key. The outcome measure for the LDST was the number of correct substitutions made in 60 s.

##### Physical activity

PA was objectively measured using the ActiGraph GT3X-BT (ActiGraph, Pensacola, FL, USA). The accelerometer was placed on the right hip with an elastic belt. Participants were asked to wear the accelerometer for seven consecutive days. However, participants were not obliged to wear the device during the night. While showering or swimming, the accelerometer had to be removed.

##### Demographic and health characteristics

Demographics and health characteristics were part of the Active Plus and Cognitive Functioning project [40]. As age, gender, educational level, marital status (living together with a spouse or living single), body mass index (BMI), and physical impairment are known to influence PA [54] and some also CF [55], these factors, assessed at baseline, were taken into account in the current study. Educational level was categorised into low (i.e., primary, basic vocational, or lower general school), moderate (i.e., medium vocational school, higher general secondary education, and preparatory academic education), or high (i.e., higher vocational school or university level) according to the Dutch educational system.

BMI was defined as the body mass (in kg) divided by the square of body height (in m). The degree of physical impairment was measured with a self-report questionnaire [56]. The participant stated for 14 common chronic illnesses (e.g., cardiovascular, osteoarthritis) and physical conditions (e.g., hearing or visually impaired) to what degree he/she was limited in his/her PA by one of the illnesses mentioned or by another illness not mentioned. For each chronic illness, the participant scored the degree of impairment on a five-point scale (0 = not applicable, 1 = not at all/hardly, 2 = a little, 3 = very, 4 = extremely). Consequently, degree of impairment was computed into three categories based on the following rules: 1) little impaired: a maximum score of 1 on at least one question; 2) medium impaired: a maximum score of 2 on at least one question; 3) very impaired: at least a score of 3 or 4 on at least one question.

##### Statistical analyses

Baseline characteristics are described for all participants who finished the randomised controlled trial using means and standard deviations for normally distributed continuous variables, medians and inter-quartile differences for non-normally distributed continuous variables, and frequency and percent for categorical variables. For further analyses, we log transformed the non-normally distributed TMT outcome measure. To assess predictors of dropout at six and 12 months, logistic regression with baseline outcome measures, demographics, and degree of impairment regarding chronic illnesses was performed and odds ratios (OR) are noted.

**Fig. 1:**
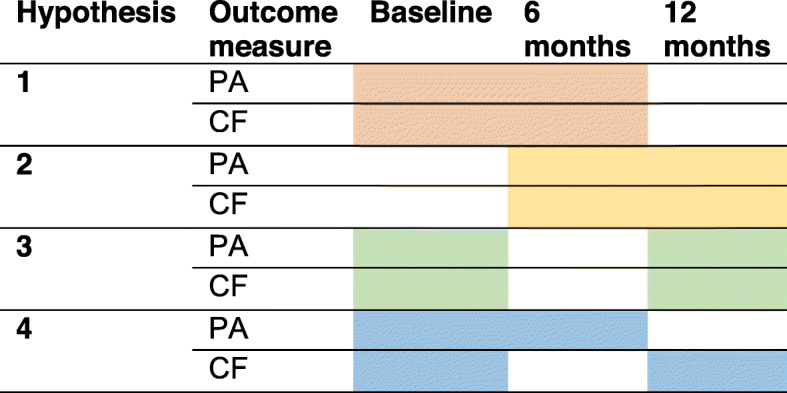
Overview of tested hypotheses. Associations between change in PA (independent variable) and change in CF (dependent variable) are tested using the data from time-points presented in colour

We tested the following longitudinal associations between PA and CF using multivariate linear regressions with the CF outcome at six or 12 months as the dependent variable and the change in PA as the independent variable (Fig. 1): 1) associations between change in PA over the first 6 months and change in CF outcomes over the same period; 2) associations between change in PA between 6 months and 12 months follow-up and change in CF outcomes over the same period; 3) associations between change in PA between baseline and 12 months follow-up and change in CF outcomes over the same period; and 4) associations of the predictive value of change in PA between baseline and 6 months follow-up for change in CF outcomes between baseline and 12 months follow-up.

Change in LPA and MVPA was calculated by subtracting the former PA score from the latter follow-up score only if scores at both time points were known. Otherwise, these scores were not taken into account in the analyses. Only valid CF tests were included in the analyses. The regressions were adjusted for age, gender, educational level, marital status, BMI, degree of impairment, baseline or 6 months follow-up CF construct score, and condition (whether the participant was part of the intervention group or the control group). Continuous covariates were standardised. To assess which PA component was the more predominant factor in relation to cognitive function, both MVPA and LPA were analysed simultaneously (in the same model). Furthermore, confidence intervals (CIs) were calculated for all outcomes. Analyses were conducted on all available and valid data without any ad hoc imputation [57]. Significance levels for all analyses were set at *p* < 0.05. All analyses were conducted using R [58].

## Results

The seven municipalities invited a total of 14,576 inhabitants, of whom 623 provided informed consent. Thirty-eight participants withdrew from the study without completing any baseline measurements. At 6 months, 19.1% (112/585) of the participants who started with the study dropped out, and at 12 months this rate was 26.2% (153/585). At both six and 12 months, drop-out was more likely for persons of a higher age (6 m: OR = 1.06, 95% CI = 1.01, 1.12, *p* = 0.027; 12 m: OR = 1.05, 95% CI = 1.00, 1.10, *p* = 0.038).

**Fig. 2:**
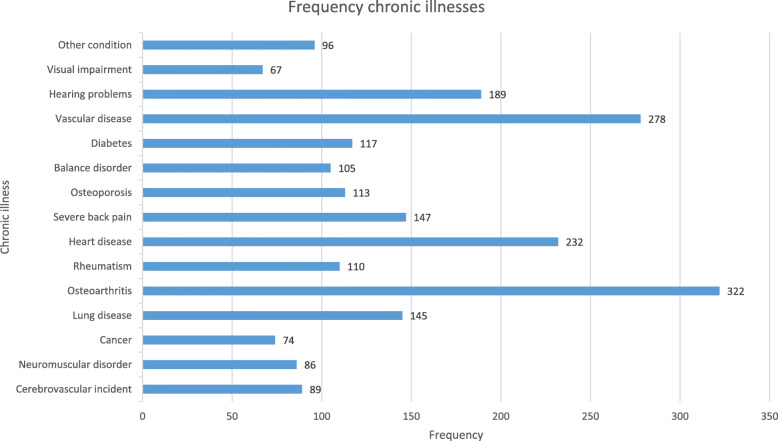
Frequency of chronic illnesses in study population. Note: participants could suffer from more than one chronic illness

As shown in Table 2, the mean age of the participants was 73.7 (±6.1) years, with 46.8% female participants. The majority of the participants were living together with a spouse (82.1%), and 48.3% were low-educated (i.e., primary, basic vocational, or lower general school). Most participants (47.7%) were medium impaired. The most frequent chronic illnesses that participants suffered from and that impaired PA were osteoarthritis (51.7% of all participants), vascular diseases (44.6%), and heart diseases (37.2%). Participants suffered from an average of 3.5 chronic illnesses or physical impairments (Fig. 2).

**Table 2:** Baseline participant characteristics (*N = 432*)

Demographic characteristics	
Age in years, mean (SD)	73.7 (6.1)
Gender, *N* (%)	
Male	230 (53.2%)
Female	202 (46.8%)
Marital status, *N* (%)	
Living single	76 (17.9%)
Living together	348 (82.1%)
Education, *N* (%)	
Low	202 (48.3%)
Moderate	89 (21.3%)
High	127 (30.4%)
Health-related characteristics	
BMI, median (IQR) ^a^	26.9 (24.3–29.4)
Degree of impairment, *N* (%)	
Little impaired	49 (11.4%)
Medium impaired	205 (47.7%)
Very impaired	176 (40.9%)
LPA in min/wk., mean (SD)	2524 (622)
MVPA in min/wk., median (IQR) ^a^	159 (66.3–292.3)
CF outcomes	
VLT – learning curve ratio, mean (SD)	1.85 (0.55)
VLT – mean no. words recalled trial 1–5, mean (SD)	7.24 (2.08)
VLT – no. words delayed recall, mean (SD)	7.57 (3.15)
TMT – time B-A in sec, median (IQR) ^a^	27.98 (12.79–49.39)
SST – SSRT in ms, mean (SD)	176.68 (98.91)
LDST – no. correct subs, mean (SD)	11.33 (4.26)

*Abbreviations*: *SD* Standard deviation, *IQR* Inter Quartile Distance, *BMI* Body mass index, *LPA* Minutes of light physical activity per week, *MVPA* Minutes of moderate-to-vigorous physical activity per week, *CF* Cognitive functioning, *VLT* Verbal learning test, *TMT* Trail making test, *SST* Stop-signal task, *SSRT* Stop-signal reaction time, *LDST* Letter digit substitution test. ^a^ non-normally distributed variables

### Associations between change in PA and change in CF over similar time periods

Table 3 shows the results of the associations between the change in PA in the first 6 months with the change in CF outcomes over the same period. An increase in LPA between baseline and 6 months follow-up was significantly positively associated with change in the mean number of words recalled in the first five trials of the VLT (coeff. = 0.18, *p* ≤ 0.01) over the same period, showing better short-term memory functions by an increase in LPA over time. Furthermore, an increase in LPA was significantly negatively associated with change in SSRT of the SST over the same period, indicating better inhibition scores after an increase in LPA (coeff. = − 9.84, *p* = 0.03). An increase in MVPA between baseline and 6 months was significantly negatively associated with change in the number of words recalled in the delayed recall trials of the VLT over the same period (coeff. = − 0.21, *p* = 0.04). In contrast to the results of LPA, this shows that an increase in MVPA over 6 months was associated with lower retention capacity. As there were no significant associations between the change in PA and CF over the 6–12-month period, nor between baseline and the 12-month period, these results are not displayed here but are added as supplementary files (Supplementary Tables 1 and 2). For further understanding, a detailed description of the PA and CF outcomes at baseline, 6 months follow-up, and 12 months follow-up can be found in Supplementary Table 3.

**Table 3:** Association between change in PA 0–6 months and change in CF over the same period ^b^

		Δ LPA 0–6				Δ MVPA 0–6			
Change in CF 0–6	***N***	coeff.	SE	95% CI	***p***	coeff.	SE	95% CI	***p***
VLT – learning curve ratio	372	0.02	0.02	−0.01;0.06	0.22	−0.00	0.02	−0.04;0.04	0.88
VLT – mean no. words recalled trial 1–5	372	0.18	0.07	0.05;0.32	**0.008**	−0.05	0.07	−0.19;0.08	0.45
VLT – no. words delayed recall	373	0.19	0.10	−0.01;0.38	0.07	−0.21	0.10	−0.41;-0.01	**0.044**
TMT – time B-A in sec ^a^	359	0.00	0.01	−0.02;0.02	0.79	−0.01	0.01	−0.03;0.02	0.62
SST – SSRT in ms	313	−9.84	4.38	−18.47;-1.21	**0.026**	−0.45	4.51	−8.42;9.32	0.92
LDST – no. correct subs	362	−0.19	0.15	−0.49;0.11	0.21	0.18	0.16	−0.13;0.49	0.26

*Abbreviations*: *PA* Physical activity, *LPA* Change in light physical activity minutes per week between 6 months follow-up and baseline, *MVPA* Change in moderate-to-vigorous physical activity minutes per week between 6 months follow-up and baseline, *SE* Standard error, *CI* Confidence interval, *ES* Effect size, *CF* Cognitive functioning, *VLT* Verbal learning test, *TMT* Trail making test, *SST* Stop-signal task, *SSRT* Stop-signal reaction time, *LDST* Letter digit substitution test. ^a^ TMT – time B-A in sec was log transformed. ^b^ Models are adjusted for baseline CF score, the covariates, and condition (control or intervention group)

### The influence of change in PA in the first six months on CF change over a year

Table 4 shows the associations of the predictive value of change in PA in the first 6 months for change in CF outcomes between baseline and 12 months follow-up. Increased LPA between baseline and 6 months follow-up was significantly positively associated with change in the mean number of words recalled in the first five trials of the VLT test over 12 months (coeff. = 0.18, *p* = 0.02), showing that an increase in LPA is of predictive value for better short-term memory functions over an extended period. No other significant relations were found.

**Table 4:** Association between change in PA 0–6 months and change in CF 0–12 months ^b^

		Δ LPA 0–6				Δ MVPA 0–6			
Change in CF 0–12	***N***	coeff.	SE	95% CI	***p***	coeff.	SE	95% CI	***p***
VLT – learning curve ratio	372	0.03	0.02	−0.01;0.06	0.18	−0.02	0.02	−0.06;0.02	0.33
VLT – mean no. words recalled trial 1–5	372	0.18	0.07	0.03;0.33	**0.016**	−0.08	0.07	−0.23;0.07	0.32
VLT – no. words delayed recall	374	0.20	0.11	−0.02;0.42	0.08	−0.00	0.11	−0.23;0.22	0.97
TMT – time B-A in sec ^a^	360	−0.01	0.01	−0.03;0.01	0.25	−0.00	0.01	−0.02;0.02	0.91
SST – SSRT in ms	306	0.98	4.28	−7.44;-9.40	0.82	−6.76	4.36	−15.35;1.82	0.12
LDST – no. correct subs	350	0.12	0.17	−0.20;0.45	0.46	−0.13	0.17	−0.47;0.20	0.44

*Abbreviations*: *PA* Physical activity, *LPA* Change in light physical activity minutes per week between six months follow-up and baseline, *MVPA* change in moderate-to-vigorous physical activity minutes per week between six months follow-up and baseline, *SE* Standard error, *CI* Confidence interval, *ES* Effect size, *CF* Cognitive functioning, *VLT* Verbal learning test, *TMT* Trail making test, *SST* Stop-signal task, *SSRT* Stop-signal reaction time, *LDST* Letter digit substitution test. ^a^ TMT – time B-A in sec was log transformed. ^b^ Models are adjusted for baseline CF score, the covariates, and condition (control or intervention group)

## Discussion

The aim of this study was to investigate the longitudinal association between change in both LPA and MVPA and the change in CF in older adults with chronic illnesses. An increase in LPA in the first 6 months was significantly associated with an increase in the same timeframe in short-term verbal memory scores and improved inhibition. In addition, the change in LPA over the first 6 months was predictive for an improved short-term verbal memory after 12 months. Interestingly, an increase in MVPA in the first 6 months was significantly associated with worse longer-term verbal memory scores (lower number of words recalled during the delayed recall trial of the VLT). No significant associations were found between change in PA and change in CF aspects between baseline and 12 months follow-up and between 6 months follow-up and 12 months follow-up.

The present study established that an increase in objectively measured LPA is beneficial for some CF aspects in older adults with chronic illnesses. The results of the few past longitudinal association studies are somewhat inconsistent [9, 59–61]. A study by Hamer et al. [59] found that PA was associated with preservation of memory and executive function over 10 years follow-up. Nonetheless, PA was measured with self-report questionnaires and the authors did not make a distinction between different intensities of PA. Lee et al. [60] did evaluate the associations between LPA and cognitive decline over an eight-year period in community-dwelling adults aged 60 and older and found that LPA was associated with a slower decline in CF after controlling for MVPA. Yet, LPA and MVPA were assessed by interviews based on a questionnaire. Stubbs et al. [9] did use accelerometers to measure PA and found that a higher level of objectively measured LPA, independent of MVPA, was prospectively associated with better cognitive ability in community-dwelling older adults. In addition, objectively assessed MVPA was also associated with better cognitive status. Although objectively assessed PA was only measured at baseline in the study by Stubbs et al., which bars the capability to examine the relationship between changes in LPA and cognitive ability, it was one of the first longitudinal studies to find that LPA is beneficial for cognitive ability in older adults. However, cognitive ability was tested with a self-report questionnaire instead of the more objective psychological test used in our study. In contrast, Zhu et al. [61] found that a dose-response relationship exists between engagement in MVPA and cognitive performance, tested with neuropsychological tests, over time. Yet, no relationship between LPA and cognitive performance over time was found. Notwithstanding, this study also only assessed PA at baseline, contrary to our study. It is suggested that this field of research needs more studies that objectively measure their outcomes.

A possible explanation for the finding that only an increase in LPA was positively associated with an improvement in CF and that an increase in MVPA was even negatively associated with change in one CF outcome can be found in the type of activities belonging to LPA and MVPA. Typical LPA activities are casually walking and household chores. Conceivably, these activities offer more opportunity for cognitive engagement with other people, listening to music, or enjoying the outdoors, and it is known that social and intellectual activities of daily life are associated with higher cognitive performance [62, 63]. Common MVPA activities, such as brisk walking or bicycling, are possibly too exhausting for further cognitive engagement, especially for older adults with chronic illnesses. However, a recent study suggests that MVPA activities that do require greater cognitive engagement, such as dancing and exercise class aerobics, do lead to greater training effects on cognition and brain connectivity than exercise requiring lower cognitive loads, such as walking briskly, in healthy elderly people [64]. Nonetheless, dancing and exercise class aerobics are generally less often executed by older adults with chronic illnesses [65, 66].

Next to cognitive aspects related to physical activities, another possible explanation could be the assumed underlying mechanisms that are responsible for changing CF through PA. A study by Voelcker-Rehage et al. [29] shows that besides cardiovascular training, other types of PA (i.e. coordination training) also improve CF of older adults. However, the mechanisms that result in these changes seem to differ depending on the intervention. Cardiovascular training was associated with increased activation of the sensorimotor network, whereas coordination training was associated with increased activation in the visual–spatial network. These differences in affected aspects of CF were also found in a recent study in rodents by Vilela et al. [67]. They showed that aerobic exercise and resistance training improved spatial working memory and hippocampal plasticity in ageing rats. However, they found that different molecular mechanisms were responsible for this. While both interventions increased neurotrophic signalling, aerobic exercise increased glutamatergic signalling and reduced DNA damage, and resistance training increased proinflammatory factors. To conclude, differences in type of PA (providing opportunity for cognitive engagement or not) and underlying molecular and neurological mechanisms related to changes in CF can explain why some aspects of CF have improved over time due to increased PA and some CF aspects did not. However, this seems too little to “justify” why there was a negative association between MVPA and long-term memory. It is clear that more research is needed in this field to affirm our findings and our possible explanation for the results.

Our findings are important in the context of the suitableness of the prescription of LPA to older adults with chronic illnesses. This population often has limited mobility and suffers from pain and fatigue [31]. As a result, older adults with chronic illnesses may be deconditioned or are not used to exercise and thus restricted to LPA only. LPA activities such as casual walking, gardening, and household chores are preferred PA activities for older adults [66, 68]. Moreover, LPA may also offer opportunities to interact with other people and thus reduce the risk of social isolation. Furthermore, increasing PA in general, especially through MVPA, is often challenging and sometimes comes along with risks of injury and deterioration as a result of physical complications. Although the PA guidelines [14] prescribe a minimum of 150 min of MVPA per week for older adults with chronic illnesses, such as type-2 diabetes, hypertension, HIV and cancer survivors, some chronic illnesses have contraindications [69] for MVPA (i.e., recent myocardial infarction, complete heart block, acute congestive heart failure, unstable angina, and uncontrolled hypertension). Increasing LPA is probably easier and, in some cases, safer for older adults [8]. Furthermore, it is probably easier to maintain in the long term. If more research confirms the results of this study, it is warranted to prescribe LPA next to MVPA for older adults with chronic illnesses to gain both health benefits and cognitive benefits. In this case, future interventions, PA guidelines, and PA programmes should address this finding.

This study has several strengths. Firstly, we objectively assessed PA with accelerometers. Although they have limitations in distinguishing between types of activities, they are considered a better measurement tool for PA than self-report questionnaires [70]. These questionnaires are prone to over-reporting and have issues with validly assessing LPA [10, 71]. Secondly, our research population is reasonably representative of the general older adult population in the Netherlands, as almost equal groups of males and females participated and most of the participants were low educated (e.g., 51%) [72]. Furthermore, BMI levels and the mean number of comorbidities (3.5) are also comparable to the general older adult population of the Netherlands [73, 74]. Therefore, these results could be generalisable to the older adults with chronic illnesses population or even to the general older adult population of the Netherlands.

This study also has some limitations. First, this study only tested the associations of change in LPA and MVPA with change in CF. To test the actual effects of change in PA on CF, a randomised controlled trial must be carried out with at least three groups (LPA intervention, MVPA intervention, control group). As our own Active Plus intervention was mainly aimed at stimulating MVPA, and we only included one experimental group in the randomised controlled trial [40], we could not test the intervention effects of change in LPA and MVPA on CF separately. In addition, isolating the independent contribution of both PA intensities is difficult. Also, it would be of great interest to include sedentary behaviour in future analyses, as it becomes more and more clear that excessive sedentary behaviour has detrimental effects on physical health [75]. Furthermore, the study period of 1 year was quite short. Future longitudinal research of longer duration is required to verify our one-year findings. Another limitation was that we performed multiple tests to analyse the associations of PA with CF. This gives a broader perspective on CF functioning instead of assessing one specific test. However, the more tests done, the more likely faulty conclusions are drawn, because the probability of a type 1 error is increased [76]. A Bonferroni correction, however, assumes that all of the hypothesis tests are statistically independent, which is not the case in the current study, as these aspects of CF are dependent. Therefore, a Bonferroni correction would be overly conservative. However, the results of this study should be considered with caution.

## Conclusions

An increase in LPA in the first 6 months was associated with better short-term verbal memory and inhibition over the same period. Furthermore, an increase in LPA in the first 6 months was a predictive value for change in short-term verbal memory over a 12-month period. MVPA, however, at the first 6 months was associated with worse longer-term verbal memory scores. It may be that LPA activities offered opportunities for PA with greater cognitive engagement than MVPA activities or that the underlying molecular and neurological mechanisms related to changes in CF differ per PA type. Thus, for older adults with chronic illnesses who may experience difficulties in being sufficiently active, an increase in LPA is probably more achievable than an increase in MVPA. In addition, an increase in LPA enhances CF more than an increase in MVPA does.

## Supplementary Information

**Additional file 1.**

**Additional file 2.**

**Additional file 3.**

## Data Availability

Study data are available from the corresponding author on reasonable request.

## Abbreviations

BMI: Body mass index
cETO: Research Ethics Committee of the Open University
CI: Confidence intervals
CF: Cognitive functioning
ES: Effect size
ICC: Intra cluster correlation
IQR: Inter quartile distance
LDST: Letter digit substitution test
LPA: Light physical activity
MMSE: Mini mental state examination
MVPA: Moderate-to-vigorous physical activity
OR: Odds-ratio
PA: Physical activity
SD: Standard deviation
SE: Standard error
SSRT: Stop-signal reaction time
SST: Stop-signal task
TMT: Trail making test
VLT: Verbal learning test
