# Supplementary material for: The longitudinal associations between change in physical activity and cognitive functioning in older adults with chronic illness (es)
Source: BMC Geriatr. 2021 Sep 4;21:478. doi: 10.1186/s12877-021-02429-x (PMC8418733; doi:10.1186/s12877-021-02429-x)
Supplement: Supplementary file 1 — Additional file 1. [file 12877_2021_2429_MOESM1_ESM.docx]

**SUPPLEMENTARY FILE 1**

**Supplementary table 1.** Association between change in PA 6-12 months and change in CF over the same period.*

|  |  | **ΔLPA 6-12** | | | | **Δ MVPA 6-12** | | | |
| --- | --- | --- | --- | --- | --- | --- | --- | --- | --- |
| **Change in CF 6-12** | ***N*** | **Coeff.** | **SE** | **95% CI** | ***p*** | **Coeff.** | **SE** | **95% CI** | ***p*** |
| VLT – learning curve ratio | 356 | -0.01 | 0.02 | -0.05;0.02 | 0.44 | 0.02 | 0.02 | -0.02;0.06 | 0.29 |
| VLT – mean no. words recalled trial 1-5 | 356 | 0.08 | 0.07 | -0.05;0.21 | 0.24 | 0.07 | 0.07 | -0.06;0.21 | 0.29 |
| VLT – no. words delayed recall | 360 | 0.02 | 0.10 | -0.18;0.22 | 0.87 | 0.15 | 0.10 | -0.06;-0.35 | 0.16 |
| TMT – time B-A in sec ^a^ | 351 | -0.01 | 0.01 | -0.03;0.01 | 0.55 | -0.00 | 0.01 | -0.02;0.02 | 0.74 |
| SST – SSRT in ms | 290 | -5.85 | 4.58 | -14.86;-3.15 | 0.20 | 1.71 | 4.73 | -7.61;11.03 | 0.72 |
| LDST – no. correct subs | 343 | -0.15 | 0.17 | -0.47;0.18 | 0.38 | 0.06 | 0.17 | -0.28;0.39 | 0.74 |

Abbreviations: PA, physical activity; LPA, change in light physical activity minutes per week between 12 months follow-up and 6 months follow-up; MVPA, change in moderate to vigorous physical activity minutes per week between 12 months follow-up and 6 months follow-up; SE, standard error; CI, confidence interval; ES, effect size; CF, cognitive functioning; VLT, verbal learning test; TMT, trail making test; SST, stop-signal task; SSRT, stop-signal reaction time; LDST, letter digit substitution test. ^a^ TMT – time B-A in sec was log transformed.* Models are adjusted for CF score at 6 months, covariates, and condition (control or intervention group).
