# Supplementary material for: The longitudinal associations between change in physical activity and cognitive functioning in older adults with chronic illness (es)
Source: BMC Geriatr. 2021 Sep 4;21:478. doi: 10.1186/s12877-021-02429-x (PMC8418733; doi:10.1186/s12877-021-02429-x)
Supplement: Supplementary file 2 — Additional file 2. [file 12877_2021_2429_MOESM2_ESM.docx]

**SUPPLEMENTARY FILE 2**

**Supplementary table 2.** Association between change in PA 0-12 months and change in CF over the same period.*

|  |  | **Δ LPA 0-12** | | | | **Δ MVPA 0-12** | | | |
| --- | --- | --- | --- | --- | --- | --- | --- | --- | --- |
| **Change in CF 0-12** | ***N*** | **Coeff.** | **SE** | **95% CI** | ***p*** | **Coeff.** | **SE** | **95% CI** | ***p*** |
| VLT – learning curve ratio | 373 | 0.01 | 0.02 | -0.02;0.05 | 0.48 | 0.02 | 0.02 | -0.01;0.05 | 0.23 |
| VLT – mean no. words recalled trial 1-5 | 373 | 0.13 | 0.08 | -0.02;0.28 | 0.10 | -0.01 | 0.07 | -0.16;0.13 | 0.85 |
| VLT – no. words delayed recall | 374 | 0.18 | 0.11 | -0.04;0.40 | 0.12 | 0.16 | 0.11 | -0.06;-0.38 | 0.15 |
| TMT – time B-A in sec ^a^ | 361 | -0.02 | 0.01 | -0.04;0.00 | 0.09 | -0.00 | 0.01 | -0.02;0.02 | 0.65 |
| SST – SSRT in ms | 304 | -4.58 | 4.46 | -13.35;-4.19 | 0.31 | -4.85 | 4.30 | -13.27;3.57 | 0.26 |
| LDST – no. correct subs | 352 | 0.13 | 0.17 | -0.19;0.46 | 0.43 | -0.12 | 0.16 | -0.43;0.20 | 0.46 |

Abbreviations: PA, physical activity; LPA, change in light physical activity minutes per week between 12 months follow-up and baseline; MVPA, change in moderate to vigorous physical activity minutes per week between 12 months follow-up and baseline; SE, standard error; CI, confidence interval; ES, effect size; CF, cognitive functioning; VLT, verbal learning test; TMT, trail making test; SST, stop-signal task; SSRT, stop-signal reaction time; LDST, letter digit substitution test. ^a^ TMT – time B-A in sec was log transformed. *Models are adjusted for baseline CF score, covariates, and condition (control or intervention group).
