# Supplementary material for: The longitudinal associations between change in physical activity and cognitive functioning in older adults with chronic illness (es)
Source: BMC Geriatr. 2021 Sep 4;21:478. doi: 10.1186/s12877-021-02429-x (PMC8418733; doi:10.1186/s12877-021-02429-x)
Supplement: Supplementary file 3 — Additional file 3. [file 12877_2021_2429_MOESM3_ESM.docx]

**SUPPLEMENTARY FILE 3**

**Supplementary table 3.** PA and CF outcomes at baseline and follow-up measurement (*N=432*).*

|  | Baseline | 6 months | 12 months |
| --- | --- | --- | --- |
| LPA in min/wk, mean (SD) | 2524 (622) | 2391 (599) | 2431 (620) |
| MVPA in min/wk, median (IQR) ꝉ | 159 (66-292) | 155 (62-278) | 146 (59-290) |
| VLT – learning curve ratio, mean (SD) | 1.85 (0.55) | 2.05 (0.56) | 2.10 (0.56) |
| VLT – mean no. words recalled trial 1-5, mean (SD) | 7.24 (2.08) | 8.21 (2.24) | 8.70 (2.38) |
| VLT – no. words delayed recall, mean (SD) | 7.57 (3.15) | 8.90 (3.20) | 9.20 (3.42) |
| TMT – time B-A in sec, median (IQR) ꝉ | 27.98 (12.79-49.39) | 23.37 (9.50-47.34) | 22.74 (11.15-45.54) |
| SST – SSRT in ms, mean (SD) | 176.68 (98.91) | 162.79 (80.30) | 155.94 (75.94) |
| LDST – no. correct subs, mean (SD) | 11.33 (4.26) | 11.76 (4.03) | 11.81 (4.33) |

Abbreviations: SD standard deviation, CF cognitive functioning, VLT, verbal learning test, TMT, trail making test; SST, stop-signal task, SSRT stop-signal reaction time, LDST letter digit substitution test. * Summary statistics using all the available and valid individual data at baseline and follow-up points ꝉ non-normally distributed variables.
